# Immune infiltrate in cancer

**DOI:** 10.18632/aging.100770

**Published:** 2015-06-25

**Authors:** Gautier Stoll, Laurence Zitvogel, Guido Kroemer

**Affiliations:** Université Paris Descartes, Sorbonne Paris Cité, Paris, France

The past decade has witnessed a major revolution in cancer research. While malignancy was (and is) generally considered as a cell-autonomous genetic and epigenetic disease, it turned out that cancer has also an immunological connotation [1]. Only if the immune system fails to eliminate the (pre-)neoplastic cells cancer can emerge and progress. During their lifetime, cancer cells are under the scrutiny of immune effectors. Only those malignant cells that ‘hide’ from the immune system or manage to actively paralyze immune effectors by immunosuppression finally manage to escape control, to invade tissues, to metastasize to distant sites and to kill their host. Importantly, successful chemotherapies and radiotherapies that prolong overall survival by years or decades (instead of weeks and months) and that act well beyond the discontinuation of the treatment generally act by reactivating the immune response against neoplastic cells, hence restoring a state of immunosurveillance [2–4]. The recent surge of immunotherapies with so-called checkpoint blockers (i.e. antibodies that neutralize the breaks that usually restrains the immune response) reinvigorates the idea that it is sufficient to unleash the forces of the immune system to obtain significant therapeutic benefit [1, 5]. Given these premises, it is not surprising that the study of the immune infiltrate that is present within the tumor either before or after therapeutic intervention can yield important biomarkers to predict the prognosis of patients with cancer, as well as their response to chemotherapy, radiotherapy or immunotherapy [1]. Recently, we started an attempt to study the immune infiltrate of several major human cancers (breast cancer, colorectal carcinoma, melanoma, non-small cell lung cancer) by means of transcriptome microarray analysis, the sole technology that yields unbiased information on the presence of distinct immune cell subtypes within the tumor bed. Notwithstanding the fact that this methodology has serious limitations (such as the loss of spatial information), it is broadly applicable across different cancer types. By using a system of ‘metagenes’ describing the co-expression of several genes for each immune subtype, we determined the composition of the immune infiltrate in close-to 3500 tumor samples from distinct cancer patients[6]. This analysis yielded important organ-specific differences in the overall composition of the immune infiltrate. More interesting, however, this type of analysis yielded important insights into the overall organization of the immune infiltrate across patient cohorts [6].

**Figure 1:**
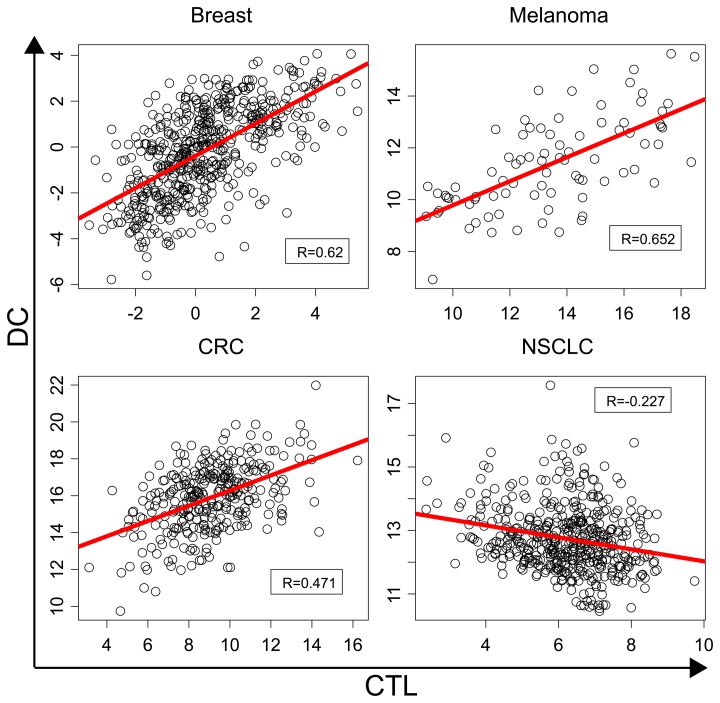
Correlation in the expression between metagenes corresponding to dendritic cells (DC) and cytotoxic T lymphocytes (CTL) in four major distinct cancer types. The levels of expression of DC and CTL metagenes were determined as arbitrary units for distinct patients (each point correspond to one patient) on logarithmic scales using the microarray cohorts described in Reference [6]. Pearson correlation coefficients (R) and linear regressions (in red) are provided for each cancer type.

We considered that the minimal ‘immune system’ within a tumor requires only two cell types, namely dendritic cells (DC) and cytotoxic T lymphocytes (CTL). DC would have to engulf portions from cancer cells to cross-present tumor-associated antigens to CTL, which in turn would attack the malignant cells, allowing for efficient tumor growth control. As a result, we determined the correlation of the expression of two metagenes, one corresponding to DC, the other corresponding to CTL, across the 3500 patient samples. Although we observed a positive correlation between the abundance of DC and CTL that are locally present in breast cancer, colorectal carcinoma and melanoma, we found a minor, though significant negative (p<0.01) correlation between the two parameters in non-small cell lung cancer patients (Fig. 1).

The aforementioned results have two major implications. First, they suggest that there are major organ-specific differences in the organization of the immune infiltrate across different cancer types. Second, in those tumors in which DC and CTL are associated among each other in a positive fashion (i.e. in breast cancer, colorectal carcinoma and melanoma), it may be sufficient to study the abundance and functional state of CTL alone as a prognostic biomarker. In contrast, in patients with non-small cell lung cancer, it may be particularly important to retrieve information on both DC and CTL to accurately predict the fate of patients[7].

Altogether, this example illustrates how important it is to comparatively study the immune infiltrate in distinct cancer types. Apparently, the overall organization of the local inflammatory and immune system can be profoundly influenced by the organ in which the tumor develops and/or the type of malignancy that arises [6]. Moreover, subtle differences in the immune context may have a profound impact on the overall probability of distinct cancer types to respond to therapeutic interventions. Is it fortuitous that non-small cell lung cancers have a rather poor response rate to all types of treatment? Or may this particular refractoriness stem from a poor organization of the local immune system?
